# Quantitative Proteomic Analysis of Hepatic Tissue of T2DM Rhesus Macaque

**DOI:** 10.1155/2017/3601708

**Published:** 2017-12-14

**Authors:** Tingfu Du, Shuaiyao Lu, Qinfang Jiang, Yun Li, Kaili Ma

**Affiliations:** ^1^Center for Drug Safety Evaluation and Research, Institute of Medical Biology, Chinese Academy of Medical Sciences, Kunming 650118, China; ^2^Medical Primate Research Center & Neuroscience Center, Chinese Academy of Medical Sciences, Beijing 100005, China; ^3^Yunnan Key Laboratory of Vaccine Research Development on Severe Infectious Diseases, Kunming 650118, China

## Abstract

Type 2 diabetes mellitus (T2DM) is a metabolic disorder that severely affects human health, but the pathogenesis of the disease remains unknown. The high-fat/high-sucrose diets combined with streptozotocin- (STZ-) induced nonhuman primate animal model of diabetes are a valuable research source of T2DM. Here, we present a study of a STZ rhesus macaque model of T2DM that utilizes quantitative iTRAQ-based proteomic method. We compared the protein profiles in the liver of STZ-treated macaques as well as age-matched healthy controls. We identified 171 proteins differentially expressed in the STZ-treated groups, about 70 of which were documented as diabetes-related gene in previous studies. Pathway analyses indicated that the biological functions of differentially expressed proteins were related to glycolysis/gluconeogenesis, fatty acid metabolism, complements, and coagulation cascades. Expression change in tryptophan metabolism pathway was also found in this study which may be associations with diabetes. This study is the first to explore genome-wide protein expression in hepatic tissue of diabetes macaque model using HPLC-Q-TOF/MS technology. In addition to providing potential T2DM biomarkers, this quantitative proteomic study may also shed insights regarding the molecular pathogenesis of T2DM.

## 1. Introduction

Type 2 diabetes mellitus (T2DM) is well known as a complex multifactorial chronic metabolic disease that is characterized by a lack of adequate insulin or insulin resistance, resulting in hyperglycemia [1, 2]. Patients with type 2 diabetes have elevated risks of many complications such as nephropathy and cardiovascular disease [3]. The incidence of T2DM is increasing at an alarming rate worldwide. However, the mechanisms that trigger the development of type 2 diabetes mellitus remain largely unknown.

Animal models have played a critical role in the exploration of disease pathophysiology and target identification and in the evaluation of prevention and treatment in vivo. To investigate the molecular and pathological mechanisms of T2DM, intraperitoneal injection of streptozotocin (STZ) rodent animal models was established [4]. These animals showed similar pathological features to T2DM, such as insulin resistance and relative insulin deficiency. Therefore, the STZ rodent models are useful animal models for the investigation of T2DM. In contrast to previous studies in rodent models [5–8], we used STZ administered to macaques via a slow intoxication protocol to produce a gradual development of liver lesions mimicking the typical, chronic evolution of T2DM in humans. The rhesus macaques have several advantages for the investigation of T2DM diseases for its genetic, morphological, physiological, and behavioral similarities to humans. Rhesus macaques in T2DM induced by the high-fat/high-sucrose diets combined with streptozotocin (STZ) in our previous study are a highly reproducible and human disease-similar model [9].

The alteration of proteins is one of the important factor that contributes to the underlying mechanism of many diseases including diabetes. Proteomic studies may be helpful to the study of the pathogenic mechanisms of T2DM and to improve current therapies [10–12]. The liver is a center of substrate and energy metabolism. It plays diverse biological roles and impacts other systems of the body which is closely linked to the pathogenesis of insulin resistance and T2DM [13, 14], but the limited availability of this tissue has been a major barrier to explore its biology. In the present study, we focus on hepatic changes in protein and gene expression associated with STZ-induced monkey T2DM using proteomic and quantitative real-time polymerase chain reaction (qRT-PCR) assay. The proteomic profile of hepatic tissue most closely reflects alterations in response to environmental stimulation and offers a novel insight into the pathology of T2DM. The discovery of proteins that are specifically altered in the T2DM liver would help us further understand the pathogenesis of T2DM.

## 2. Materials and Methods

### 2.1. Ethics Statement and Experimental Animals

The complete details of the entire study design and procedures involved were in accordance with the Declaration of Helsinki. Female rhesus macaques aged 6–8 years were from the Medical Primate Research Center of Institute of Medical Biology Chinese Academy of Medical Sciences. All animal works were approved by the Yunnan Province Experimental Animal Management Association and the Experimental Animal Ethics Committee of the Institute of Medical Biology Chinese Academy of Medical Sciences which based on the 3R principle (reduction, replacement, and refinement).

Rhesus macaques were housed in individual indoor cages under a 12 h light/dark cycle at constant temperature (24 ± 2°C). Control monkeys were provided with the basic conventional nutrients. The diet of the T2DM monkeys contained not only the basic nutrients but also with high-fat and high-sucrose diet. High-fat/high-sucrose-induced diet includes the conventional feed to offer the basic nutrients and a large dose of sucrose, animal oil, and cholesterin to assure the energy and cholesterin overload to benefit the T2DM induction. The details of the 2 diets were displayed in our previous study [9]. In the T2DM group, monkeys were fed with high-fat and high-sucrose diet for 6 months, and when hyperlipidemia and hyperinsulinemia symptom appeared, monkeys were injected with STZ at a dosage administration of 35 mg per kg of body weight by intraperitoneal injection. The clinical features of T2DM appeared after one week injection and can be sustained for a long time. Two T2DM monkeys and two control monkeys were used for proteomic assay; four T2DM monkeys and four control monkeys were used for real-time quantitative PCR validation.

### 2.2. The Assay of Glucose Tolerance Test (GTT) and Insulin Tolerance Test (ITT)

All monkeys were injected with sterilized 50% glucose solution via intraperitoneal injection at a dosage administration of 2 g per kg of body weight after fasting 16 h. For the level of blood glucose, insulin was detected at 0, 15, 30, 60, 90, and 120 min. In addition, recombined human insulin (Tonghuabao Co., China) was injected into the animal at the dose of 0.75 IU/kg body weight after fasting 16 h and the changing of blood glucose was monitored, respectively, at 0, 15, 30, 60, 90, and 120 min to survey insulin resistance. The animal was injected with 10% glucose solution immediately to avoid shock death once it emerged symptoms of low blood sugar during the test process.

### 2.3. Histopathological Evaluation

The main tissues such as pancreas, liver, and kidneys were fixed by 10% formalin neutral buffer solution. After fixation, tissues were paraffin-embedded after dehydration and cut into slices, then stained with HE staining kit (Genmed Scientifics Inc., USA) and then used haematoxylin (Genmed Scientifics Inc., USA) as a counterstaining, using the microscope to observe and analyze the lesions in all the tissue mountings.

### 2.4. Protein Preparation

Liver samples from surgery were washed in phosphate-buffered saline (PBS) solution to remove blood clots and stored in a −80°C refrigerator. Frozen liver tissues were slowly thawed in ice for the next step, and the same area from liver tissues were minced with scissors into small pieces, collected in test tubes with protein lysis buffer supplemented with 2 mM PMSF, 65 mM DTT, and protease inhibitor, and homogenized until there were no tissue pieces. The supernatant was transferred to a new tube and centrifuged at 20,000*g* for 15 min at 4°C. Total protein concentration was determined using a BCA protein assay. Bovine serum albumin (BSA) was used as a standard.

### 2.5. iTRAQ Labeling

One hundred micrograms of proteins from each sample was reduced, alkylated, and digested with trypsin (Promega). The digested peptides were then dried and reconstituted in 50 *μ*L 0.5 M triethyl ammonium bicarbonate (TEAB). Digested peptide samples were labeled using the iTRAQ kit (Applied Biosystems, Foster City, CA, USA) according to the manufacturer's protocol. The iTRAQ tags were as follows: healthy control 1-iTRAQ 114; healthy control 2-iTRAQ 115; T2DM 1-iTRAQ 116; and T2DM 2-iTRAQ 117. The labeled samples were finally combined into one sample mixture and dried with a rotary vacuum concentrator.

### 2.6. LC-MS/MS Experiments

Peptides were desalted using Zip tip C18 (Millipore, ZTC18S096, USA), concentrated by vacuum centrifuge, and stored at −20°C until further use. iTRAQ labeling was quenched by the addition of 1 M ammonium bicarbonate. Reversed-phase separation was performed on a Dionex U3000 HPLC (Dionex, Sunnyvale, CA). Durashell-C18 reverse phase column (Agela, DC952505-0) was used to purify and concentrate the labeled peptides according to the manufacturer's protocol. LC-MS/MS experiments were performed using a Bruker micrOTOF-Q III mass spectrometer (Bruker Daltonik GmbH, Leipzig, Germany) equipped with a nanospray source and Agilent 1100 high-performance liquid chromatography system (Agilent Technologies, Livermore, CA). The samples were acquired in positive and high-sensitivity mode using electrospray ionization (ESI) method. Peptide sequences were identified from MS/MS fragmentation spectra using the Mascot search engine (Matrix Science, UK). The matched peptides that were considered as potential candidates had the highest Mascot score (≥65) and a peptide sequence coverage of 20 percent of the matched peptide.

The original data were then analyzed using Perseus software (version 1.3.0.4). Proteins whose levels differed by ≥1.2 or ≤0.8 times compared with the controls were used to define differential protein expression.

### 2.7. Pathway Analysis

Functional annotation clustering of selected genes was performed using the DAVID Bioinformatics Resources database. The differential expression proteins identified in the hepatic tissue were used as “background” for GO enrichment of significantly (*t*-test *p* value < 0.05) expressed proteins. Gene enrichment (enrichment *p* value) of each pathway was analyzed, and the pathways with *p* < 0.01 were listed and considered significantly different.

### 2.8. Validation of Differentially Expressed Proteins by Quantitative Real-Time PCR

The proteomic data were validated utilizing SYBR Green-based real-time quantitative PCR (qPCR) performed in 96-well plates on CFX96 real-time PCR detection system (Bio-Rad, Hercules, CA). The liver total RNA samples from the rhesus monkey were analyzed. Proteins with high fold change (≥2.0 or ≤0.5) in T2DM monkeys were selected for validation. Actin was used as a reference gene because it was the most stably expressed gene from T2DM. 500 ng of total RNA from each sample was retrotranscribed to cDNA (Eastep® RT Master Mix (5x) Kits, LS2054, Promega, USA). qPCR was run on Eastep qPCR Master Mix (2x) (LS2068, Promega, USA) according to the manufacturer's recommendations. All of the genes were performed in triplicates. Primers were seen in Table 1. The comparative Ct (2^−ΔΔCt^) method was used to quantify expression of genes, and fold change (FC) was used to present data.

### 2.9. Statistics Analysis

All statistics were performed using the GraphPad Prism software (GraphPad). Student's *t*-test was used in this article. A value of *p* < 0.05 was considered significant.

## 3. Results

### 3.1. Results of GTT and ITT

**Figure 1:**
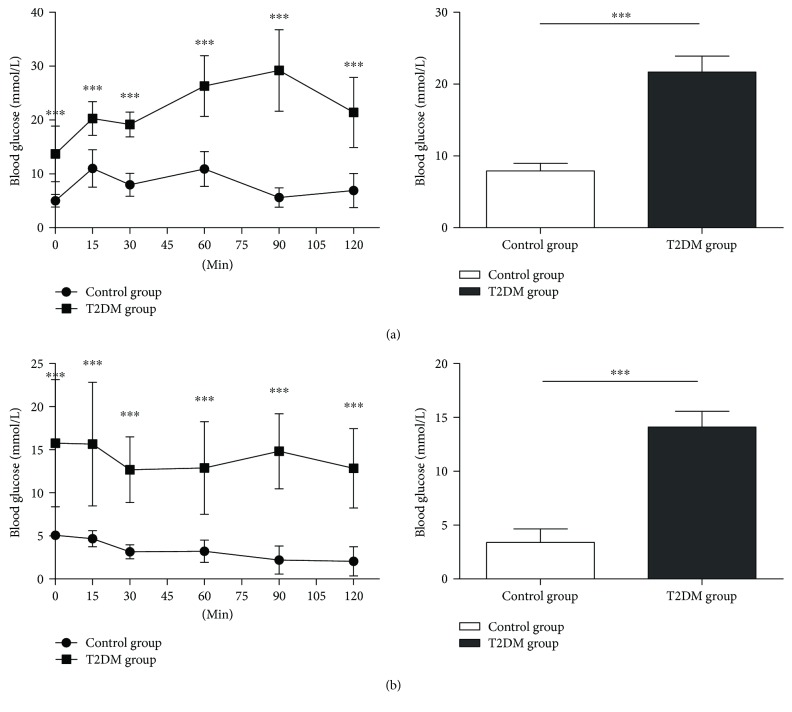
The changing level of blood glucose revealed by GTT and ITT. (a) The results of the glucose tolerance test (GTT) showed that the release of blood glucose in the T2DM group was at a higher level and the blood glucose value of 2-hour post meal was apparently greater than 11.1 mmol/L, and the AUC (area under ROC curve) analysis indicated that the GTT change curve was extremely significantly different from the control group. (b) The results of the insulin tolerance test (ITT) showed that the release of blood glucose in the T2DM group was at a higher level after intraperitoneal injection of recombinant human insulin, and AUC analysis indicated that the ITT change curve was extremely significantly different between the T2DM group and the control group. ^∗∗∗^ *p* < 0.001. Error bars indicate SD. Control (*n* = 6); T2DM (*n* = 6).

The results of glucose tolerance test (GTT) showed that the release of blood glucose in the T2DM group was at a higher level and the blood glucose value of 2-hour post meal was apparently greater than 11.1 mmol/L, and the AUC (area under the ROC curve) analysis indicated that the GTT change curve was extremely significantly different from the control group (*p* < 0.001) (Figure 1(a)). The results of the insulin tolerance test (ITT) showed that the release of blood glucose in the T2DM group was at a higher level after intraperitoneal injection of recombinant human insulin, and AUC analysis indicated that the ITT change curve was extremely significantly different between the T2DM group and the control group (*p* < 0.001) (Figure 1(b)).

### 3.2. Results of Histopathology

**Figure 2:**
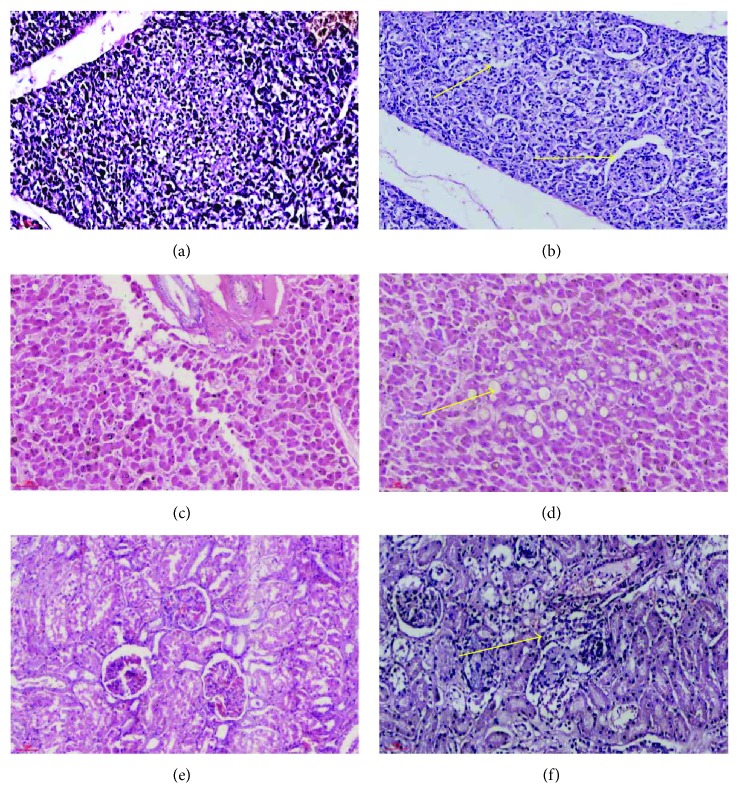
Histopathological detection of the pancreas, liver, and kidneys stained with HE. The results of HE staining in some tissues showed that the pancreatic islet cells were absent in the T2DM rhesus macaques, and the islet atrophy was observed. (a) Normal pancreatic tissue. (b) Diabetic pancreatic tissue. Liver tissues showed fat vacuoles, and hepatic cell fatty degeneration was obvious. (c) Normal liver tissue. (d) Diabetes liver tissue. The renal tissue has interstitial chronic inflammatory cell infiltration. The histopathological features of T2DM were found in all tissues. However, no obvious abnormal lesions were found in the normal control group. (e) Normal kidney tissue. (f) Diabetes kidney tissue.

The results of HE staining in some tissues showed that the pancreatic islet cells were absent in the T2DM rhesus macaques, and the islet atrophy was observed (Figures 2(a) and 2(b)). Liver tissues showed fat vacuoles, and hepatic cell fatty degeneration was obvious (Figures 2(c) and 2(d)). The renal tissue has interstitial chronic inflammatory cell infiltration. The histopathological features of T2DM were found in all tissues. However, no obvious abnormal lesions were found in the normal control group (Figures 2(e) and 2(f)).

### 3.3. Analysis of Differentially Expressed Proteins

Mass spectrometry-based proteomics was applied to samples of the liver in mice with STZ-treated monkeys. The results have shown that 813 proteins are identified. We identified 171 proteins differentially expressed in the STZ-treated groups (Table S 1, supplementary data), about 70 of which were documented as diabetes-related gene in previous studies. Sixteen proteins of them whose levels differed by ≥2.0 or ≤0.5 times compared with the control were shown in Table 2.

### 3.4. Real-Time Quantitative PCR Validation

**Figure 3:**
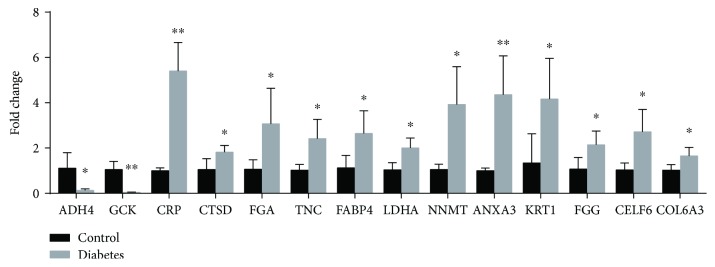
Quantitative PCR validation of proteomic data. Fourteen differentially expressed proteins were validated by qPCR. The Student's *t*-test was used to determine *p* values (^∗^ *p* < 0.05, ^∗∗^ *p* < 0.01). Error bars indicate SD. Control (*n* = 4); T2DM (*n* = 4).

Sixteen proteins with high fold change (≥2.0 or ≤0.5) were selected for validation. Among those proteins, MPO and CA1 failed qPCR detection for technical reasons, so those two genes were removed from further analysis. Fourteen differentially expressed proteins were validated by qPCR (Figure 3). The data supported a strong consistency between the qPCR result and proteomic data. Primers used in this article are shown in Table 1.

### 3.5. Pathway Analysis

DAVID web service was used for functional annotation. The results of the bioinformatic analysis are shown in Table 3. As shown in Table 3, most of the proteins are associated with the glycolysis/gluconeogenesis, fatty acid metabolism, complements and coagulation cascades, and so on.

## 4. Discussion

Many researches have reported that the main characters of the animal models of T2DM are high blood glucose and high-insulin hematic disease [15]. However, small experimental animals fail to simulate the traditional and complete clinical features of the patients who suffer T2DM [16]. It is hard to build a model that emerges simultaneously all features of obesity complicated with hyperlipidemia, insulin resistance, mild high blood glucose, and high-insulin hematic disease. Thus, designing a reasonable high-fat/high-sucrose diet and the dose of the STZ seems to be more important [17, 18]. Rhesus macaques as advanced nonhuman primates share high homology and similarity in genetic, physiological, and biochemical aspects with human and can simulate the occurrence and development of human disease [19]. In our previous study, we have explored the experimental condition of high-fat/high-sucrose diet in combination with the low dose of the STZ to induce T2DM [9]. The results showed that the monkeys fed with high sugar and high fat for 6 months began to appear hyperlipidemia and hyperinsulinemia, but their fasting glucose was within the normal range (<6.8 mmol/L). Even if the monkeys are fed with high sugar and high fat for one year, their fasting glucose was still at a normal level. The monkeys were injected with STZ at a dosage administration of 35 mg/kg of body weight as soon as hyperlipidemia and hyperinsulinemia symptom appeared. The monkeys showed clinical features of T2DM after one week injection, and those features can be sustained for a long time. We have also made some research on T1DM monkeys (the data were not shown in this study). In the T1DM group, monkeys were intravenously injected with STZ at dosage administration of 50 mg/kg of body weight for 1 to 3 times or intraperitoneal injection at dosage administration of 100 mg/kg body weight for 1 to 3 times. The clinical features of T1DM appeared after one week injection, but the monkeys cannot survive for a long time, insulin need to be injected to sustain life. In this study, we built the rhesus macaque model of T2DM successfully. The rhesus macaque models we have built could simulate the clinical manifestations and pathological features of the T2DM in humans through comprehensive analysis and evaluation [9]. Analysis and assessment of the rhesus macaque T2DM models from the clinical features and parts of histopathologic features show that the results match basically the diagnostic criteria of the human T2DM. Both insulin tolerance test and insulin resistance test suggest that the monkeys in T2DM group possess key clinical features with human T2DM patients. The histopathological lesion was found in pancreas islet and hepatic tissue. Steatosis was found severely in the liver cells in the T2DM group, which is a risk factor in the development of T2DM [20–22]. The results indicate that the monkeys in the T2DM model group not only have similar characters with humans in the physiological responses but also possess basically clinical diagnosis feature of human T2DM.

Hepatic tissue plays an important role in T2DM [23, 24]. It is characterized by impaired suppression of hepatic gluconeogenesis and glucose output by insulin, which plays a crucial role in the pathogenesis of hyperglycemia and glucose intolerance [25, 26]. But the molecular mechanisms of the liver in T2DM are still unclear. In the present study, we integrate high-resolution, high mass accuracy mass spectrometry and proteomic technologies for an unbiased discovery and verification of hepatic tissue-specific proteomic changes in nonhuman primate animal model of diabetes. 174 liver tissue proteins with functional relevance to T2DM were found to be significantly differentially expressed compared to control. We found that these proteins are mainly involved in glycolysis/gluconeogenesis, fatty acid metabolism, complements and coagulation cascades, and so on. It indicates that our T2DM monkeys are very similar to human diabetes as these pathways have previously been reported associations with diabetes. These pathways have been linked to T2DM pathogenesis and could serve as protein biomarkers. In addition, the other pathway may offer new insight into the pathogenesis of T2DM, such as tryptophan metabolism pathway. It is commonly accepted that T2DM is associated with altered metabolic status [27, 28]. Some metabolites such as aromatic amino acid metabolite are closely correlated to diabetes [28, 29]. Tryptophan metabolism within different tissues is associated with numerous physiological functions. The liver regulates tryptophan homeostasis through degrading tryptophan in excess. Tryptophan plays a crucial role in the regulation of growth and feed intake, mood and behavior, and immune responses [30]. It is reported that tryptophan metabolism in the plasma is associated with the development of T2DM [31, 32]. Our results have shown that tryptophan metabolism pathway in hepatic tissue is also closely related to the occurrence of the T2DM which may help us knowing about the pathogenesis of T2DM further.

Some differentially expressed proteins with high fold change were selected in this study to discuss as previous studies have shown associations of T2DM, such as GCK, CRP, CAT, FABP4, MPO, and LDHA. The GCK gene encodes glucokinase, which catalyzes the first step in most glucose metabolism pathways, to maintain glucose homeostasis in the beta-cells of the pancreas [33]. Previous work has indicated that GCK activity is decreased in type 2 diabetes [34, 35]. Consistent with prior reports, we find that the expression of GCK in T2DM liver in our study is also decreased. It indicates that defective hepatic GCK is sufficient to impair glycogen synthesis and increased gluconeogenesis was established in previous studies [36–38]. Previous study have report that GCK is regulated by posttranslational and transcriptional mechanisms in diabetes [39]. Epigenetic modification of the GCK gene was also shown to influence the onset of diabetes [40]. Overall, these data suggest that progressive worsening of type 2 diabetes is significantly associated with suppression of GCK. C-reactive protein (CRP) is an acute phase protein that is found in blood plasma and secreted by the liver or adipose tissue, and its level rises in response to inflammation. The primary biological function of CRP is recruiting the complement system and macrophages to mediate the elimination of pathogens and the host necrotic cells [41, 42]. CRP protein is significantly increased in the presence of infection and inflammation. The activation of inflammatory could stimulate an increase in insulin resistance [43]. It has been reported that CRP could increase the risk of diabetes [44, 45]. Catalase (CAT) is an enzyme that converts hydrogen peroxide to water and molecular oxygen. This enzyme is located within cells in peroxisomes and is most abundant in erythrocytes, hepatocytes, and nephrons [46]. Ozkul et al. reported that the plasma level of CAT was lower in T2DM patients with neuropathy [47]. CAT and SOD activities were significantly decreased in T2DM compared with the control subjects [48]. Fatty acid-binding protein 4 (FABP4) is abundantly expressed in adipocytes and plays important roles in adipocyte differentiation and lipid metabolism [49]. Multiple studies have been showed that FABP4 functions in the free fatty acid transport, regulating whole body insulin sensitivity [50, 51]. A higher serum FABP4 level was found in T2DM [52]. It was also higher in the liver tissue of T2DM than in the control group [53]. Myeloperoxidase (MPO) participates in developing of inflammation [54]. There has been a study about MPO expressed higher in T2DM [55]. There is evidence that oxidative stress plays an important role in the etiology and progression of diabetes [55]. Obesity and type 2 diabetes are known to be associated with increased inflammation. Lactic acid dehydrogenase (LDHA) encoding enzymes involved in pyruvate metabolism. Pyruvate metabolism was shown to be implicated in diabetes. In general, glycolytic pathway was shown to be dysregulated in diabetes. Islets of individuals with diabetes display an increase in the expression of LDHA when compared to controls [56]. LHDA showed crucial importance as overexpression of LDHA in insulin-secreting β-cells affects glucose-induced insulin secretion. Islets of individuals with diabetes also display an increase in the expression of LDHA [57].

In conclusion, those selected proteins in our study are quite consistent with earlier studies, thereby supporting the role they play in the molecular pathogenesis of T2DM.

The differentially expressed proteins and pathways in our study suggested that fundamental metabolic, insulin signaling and hepatic tissue inflammation in the liver may be involved in the course of T2DM pathogenesis. In summary, our approach successfully identified a protein profile in nonhuman primates that can help us further understand the pathogenesis of T2DM.

## Figures and Tables

**Table 1:** Primers were used for quantitative PCR validation in this study.

Gene	Forward primer	Reverse primer
ADH4	AGTTCGCATTCAGATCATTGCT	CTGGCCCAATACTTTCCACAA
GCK	CCTGGGTGGCACCAACTTCAG	TAGTCGAAGAGCATCTCAGCA
CELF6	AGTTTGGTGATGCGGAACTCA	CATTGCCTGAATAGCAGTCTG
ANXA3	TCAGCCCATCAGTGGATGCTG	CTGTGCATTTGACCTCTCAGT
CTSD	ATTCAGGGCGAGTACATGATCC	CGACACCTTGAGCGTGTAG
TNC	TCCCAGTGTTCGGTGGATCT	TTGATGCGATGTGTGAACACA
COL6A3	CTGTTCCTCTTTGACGGCTCA	CCTTGACATCATCGCTGTACTGA
KRT1	AGTGCTTATATGACCAAGGTGG	ATGCTGTCCAGGTCGAGACT
FGA	TGTCGAGGGTCATGCAGTAG	CAAGTTGCTTCTGCTGATCTTCA
FGG	AGACACGGTGCAAATCCATGA	GCCCGCTCTGTTTAGCTCC
LDHA	ATGGCAACTCTCAAGGATCAGC	CCAACCCCAACAACTGTAATCT
NNMT	TGGTGACCTATGTGTGTGATCT	CCCTGGCTTCAGTAGGCTG
FABP4	ACTGGGTCAGGAATTTGACG	CTGGTGGAAGTGACGCCTT
CRP	GTCACAGTAGCTCCAGTACACA	AAAGCTCCCACCGAAGGAATC

**Table 2:** Differentially expressed proteins in T2DM macaques that were selected using a cutoff point of fold change ≥ 2 or ≤0.5.

Gene name	Description	Accession number	Fold change
ADH4	Alcohol dehydrogenase 4	F7GMI7	0.45
GCK	Glucokinase	F6PLG6	0.50
MPO	Myeloperoxidase	F7BAA9	2.00
CELF6	CUGBP Elav-like family member 6	F7GCB0	2.00
ANXA3	Annexin (fragment)	F6Z8Y2	2.00
CTSD	Cathepsin D preproprotein	F7H7Y3	2.05
TNC	Tenascin C	F7ECK5	2.06
CA1	Carbonic anhydrase 1	F7CLQ5	2.09
COL6A3	Collagen alpha-3(VI) chain isoform 4	I2CWG4	2.17
KRT1	Keratin 1	F7B777	2.17
FGA	Fibrinogen alpha chain	F6UZ60	2.22
FGG	Fibrinogen gamma	F6UYY8	2.39
LDHA	L-lactate dehydrogenase	H9ES85	2.43
NNMT	Nicotinamide N-methyltransferase	F7ERX8	2.48
FABP4	Adipocyte-type fatty acid-binding protein	F7GLY0	3.00
CRP	C-reactive protein	F7DHQ1	3.47

**Table 3:** The enriched terms (or function) of differential expression genes.

Category	Term	Count	*p* value
KEGG_PATHWAY	hsa00010: glycolysis/gluconeogenesis	10	3.940*E*−05
KEGG_PATHWAY	hsa00071: fatty acid metabolism	8	1.070*E*−04
KEGG_PATHWAY	hsa04610: complement and coagulation cascades	9	6.610*E*−04
KEGG_PATHWAY	hsa00380: tryptophan metabolism	7	8.060*E*−04
KEGG_PATHWAY	hsa00680: methane metabolism	3	1.109*E*−02
KEGG_PATHWAY	hsa04614: renin-angiotensin system	4	1.130*E*−02
KEGG_PATHWAY	hsa00620: pyruvate metabolism	5	2.559*E*−02
KEGG_PATHWAY	hsa00280: valine, leucine, and isoleucine degradation	5	3.488*E*−02
KEGG_PATHWAY	hsa00520: amino sugar and nucleotide sugar metabolism	5	3.488*E*−02

## Supplementary Materials

Supplementary Materials Table S1: Profile of differentially expressed proteins.
